# Downregulation of circ_0037655 impedes glioma formation and metastasis via the regulation of miR-1229-3p/ITGB8 axis

**DOI:** 10.1515/biol-2021-0048

**Published:** 2021-05-10

**Authors:** Wenhui Zou, Yalei Cao, Kai Cheng, Changyu Li, Fu Zhu, Shumao Yang, Maolin Jin, Shaojun Song

**Affiliations:** Department of neurosurgery, Hainan Cancer Hospital, No. 6, Changbin Road, Haikou City, Hainan Province, 570100, China

**Keywords:** circ_0037655, glioma, miR-1229-3p, ITGB8

## Abstract

**Background:**

Glioma is the most frequent, highly aggressive primary intracranial malignant tumor. Circular RNA (circRNA) circ_0037655 has been reported to be a vital regulator in glioma. The different functional mechanism behind circ_0037655 was investigated in the current study.

**Methods:**

The expression of circ_0037655, microRNA-1229-3p (miR-1229-3p) and integrin beta-8 (ITGB8) was detected via the quantitative reverse transcription-polymerase chain reaction (qRT-PCR). Cellular research was performed via colony formation assay for cell proliferation, flow cytometry for cell cycle and cell apoptosis, scratch assay for cell migration, as well as transwell assay for cell migration and invasion. Western blot was used for detection of ITGB8 protein and epithelial–mesenchymal transition (EMT) process. Dual-luciferase reporter assay was implemented for the binding analysis of potential targets. *In vivo* assay was administered via xenograft in mice.

**Results:**

Upregulation of circ_0037655 was affirmed in glioma samples and cells. Tumor formation and metastasis of glioma were inhibited after circ_0037655 was downregulated. miR-1229-3p acted as a target of circ_0037655, and its upregulation was responsible for the function of si-circ_0037655 in glioma cells. miR-1229-3p functioned as a tumor inhibitor in glioma progression by targeting ITGB8. circ_0037655 modulated the ITGB8 expression by targeting miR-1229-3p. *In vivo* knockdown of circ_0037655 also suppressed glioma tumorigenesis by acting on the miR-1229-3p/ITGB8 axis.

**Conclusion:**

This study showed that downregulation of the expression of circ_0037655 could inhibit glioma progression by acting on the miR-1229-3p/ITGB8 axis. The specific circ_0037655/miR-1229-3p/ITGB8 axis was disclosed in glioma research.

## 1 Introduction

Glioma remains the most dangerous brain tumor, and the prognostic results for most patients are quite poor [1]. Surgical removal followed by radiotherapy and adjuvant chemotherapy is the current standard treatment for glioma patients [2]. The therapeutic resistance and tumor relapse have limited the therapeutic outcomes, and the targeted therapy has been found to optimize the treatment of glioma [3]. Circular RNAs (circRNAs) and microRNAs (miRNAs) that belong to two different families of non-coding RNAs (ncRNAs) have great values in glioma therapy [4,5].

circRNAs are known as the covalent closed-loop structures after non-classical backsplicing of linear pre-messenger RNAs (pre-mRNAs) [6]. Increasing studies indicated that circRNAs affected the developing processes of tumors by serving as the sponges of short miRNAs to mediate the gene expression [7,8]. Zhang et al. identified that circTRIM33-12 acted as a sponge of miR-191 to inhibit the progression of hepatocellular carcinoma by upregulating the TET1 expression [9]; circRNA cTFRC was found to promote the malignant progression of bladder carcinoma via relieving the TFRC expression inhibition caused by miR-107 [10]; and hsa_circ_001895 functioned as a tumor promoter in clear cell renal cell carcinoma through miR-296-5p-controlled SOX12 regulation [11].

In glioma, circ_0034642 increased the BATF3 expression to generate the tumorigenic function via targeting miR-1205 [12] and circ_0076248 facilitated the carcinogenesis by the miR-181a/SIRT1 pathway [13]. hsa_circ_0037655 (circ_0037655) is a novel circRNA from host gene CREB-binding protein (CREBBP). Qiao et al. have unraveled that the promoting effect of circ_0037655 on glioma development was partly associated with the miR-214/PI3K axis [14]. Nevertheless, the other mechanism underlying circ_0037655 in regulating the glioma progression remains to be explored.

microRNA-1229-3p (miR-1229-3p) has abnormal expression and prognostic significance in gastric cancer [15], as well as in head and neck squamous cell carcinoma [16]. Cao et al. reported that the function of circ_0037251 knockdown in glioma was achieved by inhibiting the binding of miR-1229-3p and the 3′-untranslated region (3′-UTR) of mTOR [17]. In addition, integrin beta-8 (ITGB8) is a commonly researched regulator in human tumors, including glioma [18]. The signal networks of circ-TTBK2/miR-761/ITGB8 [19] and circ_0046701/miR-142-3p/ITGB8 [20] have been discovered in glioma reports. This study was performed to study whether the regulation of circ_0037655 in glioma was related to miR-1229-3p and ITGB8, intending to provide a better understanding of the functional mechanisms of circ_0037655.

## 2 Materials and methods

### 2.1 Tissue acquisition

A total of 35 paired glioma and normal brain tissue specimens were, respectively, acquired from patients with glioma (*n* = 35) and intracranial injury (*n* = 35) at Hainan Cancer Hospital, followed by the short-term preservation in liquid nitrogen.


**Informed consent:** Informed consent has been obtained from all individuals included in this study.
**Ethical approval:** The research related to human use has been complied with all the relevant national regulations, institutional policies and in accordance with the tenets of the Helsinki Declaration and has been approved by the Ethics Committee of Hainan Cancer Hospital.

### 2.2 Cell culture

Human glioma cell lines (T98G and LN229) and normal astrocyte NHA from QCHENG BIO (Shanghai, China) were washed with phosphate buffer solution (PBS; Gibco, Carlsbad, CA, USA) and digested in 0.25% Trypsin (Gibco). Fresh medium was prepared by adding 10% fetal bovine serum (FBS; Gibco) and 1% penicillin/streptomycin solution (Gibco) into the basic Dulbecco’s modified Eagle medium (DMEM; Gibco). The conditions of cell culture were the temperature of 37°C, the gaseous phase of 95% air and 5% CO_2_ and the humidity of 80%.

### 2.3 Transfection of oligonucleotides or vectors

Oligonucleotides including small interfering RNA (siRNA) against circ_0037655 (si-circ_0037655), miR-1229-3p mimic, miR-1229-3p inhibitor and their negative controls (si-NC, miR-NC mimic and miR-NC inhibitor) were directly purchased from GenePharma (Shanghai, China). Vectors of shRNA hairpin RNA (shRNA) against circ_0037655 and the negative control (sh-circ_0037655 and sh-NC), overexpression vector pCE-RB-Mam-circ_0037655 (oe-circ_0037655) and the basic pCE-RB-Mam vector (oe-NC) were obtained from RiboBio (Guangzhou, China). ITGB8 sequence was cloned into the pcDNA vector (pcDNA-NC; Invitrogen, Carlsbad, CA, USA) to generate the pcDNA-ITGB8. Transfection was performed in T98G and LN229 cells using Lipofectamine™ 3000 (Invitrogen), according to the provided guidelines for users.

### 2.4 Quantitative reverse transcription-polymerase chain reaction (qRT-PCR)

The extraction of total RNA was conducted from tissue samples and cell lines using TRIzol™ Plus RNA Purification Kit (Invitrogen). RevertAid RT Reverse Transcription Kit (Thermo Fisher Scientific, Waltham, MA, USA) was applied for reverse transcription, followed by the expression detection by EXPRESS One-Step Superscript™ qRT-PCR Kit (Invitrogen) in compliance with the manufacturer’s directions. Total RNA sample was treated with Ribonuclease R (RNase R; Epicentre Technologies, Madison, WI, USA) at 37°C, then qRT-PCR detection of circ_0037655 and CREBBP was carried out 30 min later. The primer sequences for the objective genes are shown as follows: circ_0037655, forward (F): 5′-AGGTTTTTGTCCGAGTGGTG-3′ and reverse (R): 5′-TCACCCAGGGTCACATTCTC-3′; CREBBP, F: 5′-CGTGTCACAGGGACAGGTG-3′ and R: 5′-TGTCGTGTGCTGGAGAGATG-3′; miR-1229-3p, F: 5′-CCACTGCCCTCCCA-3′ and R: 5′-GGTCCAGTTTTTTTTTTTTTTTCTGT-3′; ITGB8, F: 5′-CTGAAGAAATATCCTGTGGA-3′ and R: 5′-ATGGGGAGGCATGCAGTCT-3′; β-actin, F: 5′-GTGGCCGAGGACTTTGATTG-3′ and R: 5′-CCTGTAACAACGCATCTCATATT-3′; U6, F: 5′-CTCGCTTCGGCAGCACA-3′ and R: 5′-AACGCTTCACGAATTTGCGT-3′. U6 was used for standardizing the expression of miR-1229-3p and the other levels were normalized by β-actin. Data were analyzed by the comparative cycle threshold (2^−∆∆Ct^) method to acquire the relative expression levels.

### 2.5 Colony formation assay

After transfection for 24 h, 3 × 10^2^ T98G and LN229 cells were seeded into the 12-well plates to be cultured for 14 days in the 37°C incubator with 5% CO_2_. After the fixation and staining of these colonies in 4% paraformaldehyde and 0.5% crystal violet (Sigma-Aldrich, St. Louis, MO, USA), cell proliferative ability was assessed by counting the number of colonies.

### 2.6 Flow cytometry

Flow cytometry was exploited for the determination of cell cycle and cell apoptosis. T98G and LN229 cells were stained using propidium iodide (PI) of Cell Cycle Assay Kit (Dojindo, Kumamoto, Japan) and Annexin V-fluorescein isothiocyanate (FITC)/PI of Annexin V-FITC/PI Apoptosis Detection Kit (Dojindo) referring to the standard operating procedures. Cell detection was then performed by flow cytometer (BD Biosciences, San Diego, CA, USA).

### 2.7 Scratch assay

Two paralleled scratches were made by a sterile 200 μL pipette tip in the single-layer T98G and LN229 cells. PBS (Gibco) was used to remove the scraped cells, and the remaining cells were cultivated for 24 h in normal cell medium. Cell pictures at 0 and 24 h were obtained, and the migration distance (scratch width_(0 h)_ − scratch width_(24 h)_) of the experimental group was calculated relative to that of the control group (set as 1).

### 2.8 Transwell assay

Cell migration and invasion were, respectively, examined in a 24-well transwell chamber (Corning Inc., Corning, NY, USA) and a transwell chamber packaged with matrigel (Corning Inc.). A total of 1 × 10^5^ cells in serum-free medium were seeded into the top chamber and the bottom chamber was filled with 600 µL of 10% FBS + DMEM. After 24 h, the migrated and invaded cells into the bottom chamber were fixed and stained by 4% paraformaldehyde and 0.5% crystal violet (Sigma-Aldrich). Cell images were acquired under the inverted microscope (Olympus, Tokyo, Japan) of 100× magnification, followed by counting the cells in three fields of view.

### 2.9 Western blot

Radioimmunoprecipitation assay Lysis and Extraction Buffer (Thermo Fisher Scientific) was employed for extracting total proteins. Western blot analysis was performed as previously described [21]. Antibodies (Santa Cruz Biotechnology, Santa Cruz, CA, USA) used for this study contained the primary antibodies against E-cadherin (sc-8426, 1:1,000), Vimentin (sc-6260, 1:1,000), ITGB8 (sc-514150, 1:1,000), internal control β-actin (sc-47778, 1:1,000) and the secondary antibody goat anti-mouse IgG-HRP (sc-2005, 1:5,000). Immunoreactive bands were determined by the Western Blotting Luminol Reagent (Santa Cruz Biotechnology), and protein level was quantified via Image Pro Plus 6.0 image analysis software (Bio-Rad, Hercules, CA, USA).

### 2.10 Dual-luciferase reporter assay

The wild-type (WT) luciferase plasmids were constructed by inserting the sequences of circ_0037655 and ITGB8 3′-UTR (with the binding sites of miR-1229-3p) into the pGL-3 control vector (Promega, Madison, WI, USA). The WT plasmids were defined as WT-circ_0037655 and WT-ITGB8. Meanwhile, the binding sites for miR-1229-3p in circ_0037655 and ITGB8 3′-UTR sequences were mutated to construct the mutant-type (MUT) luciferase plasmids MUT-circ_0037655 and MUT-ITGB8. These plasmids were, respectively, transfected into T98G and LN229 cells with miR-1229-3p mimic or miR-NC mimic for 48 h. Ultimately, the relative luciferase activity was measured by the Dual-Luciferase Reporter Assay System (Promega) following the manufacturer’s guidelines.

### 2.11 Xenograft in mice

Ten BALB/C male nude mice (5–6-week old, 20–25 g) were bought from Vital River Laboratory Animal Technology Co. Ltd (Beijing, China), then subcutaneously injected with 2 × 10^6^ LN229 cells with stable transfection of sh-circ_0037655 or sh-NC (5 mice/group) at Hainan Cancer Hospital. After cell injection for 1 week, tumor indicators (including length and width) were monitored by a digital caliper and tumor volume was calculated using the formula: length × width^2^ × 0.5. Four weeks later, tumors were weighed after mice were euthanatized via the CO_2_ asphyxia method. Then circ_0037655 and miR-1229-3p levels in tumors were assayed by qRT-PCR, while the ITGB8 protein expression was determined using western blot.


**Ethical approval:** The research related to animal use has been complied with all the relevant national regulations and institutional policies for the care and use of animals and has been approved by the Animal Ethics Committee of Hainan Cancer Hospital and followed the Welfare Guidelines for Laboratory Animals of National Institutes of Health (NIH).

### 2.12 Statistical analysis

All experiments were performed three times and data were expressed as mean ± standard deviation. SPSS 24.0 and GraphPad Prism 7 were used for data analysis and figure generation. The comparison of difference was conducted using Student’s *t*-test and one-way analysis of variance followed by Tukey’s test, respectively, for two groups and multiple groups. In general, *P* < 0.05 was deemed as the significant difference.

## 3 Results

### 3.1 circ_0037655 was dysregulated with a high level in glioma samples and cells

**Figure 1:**
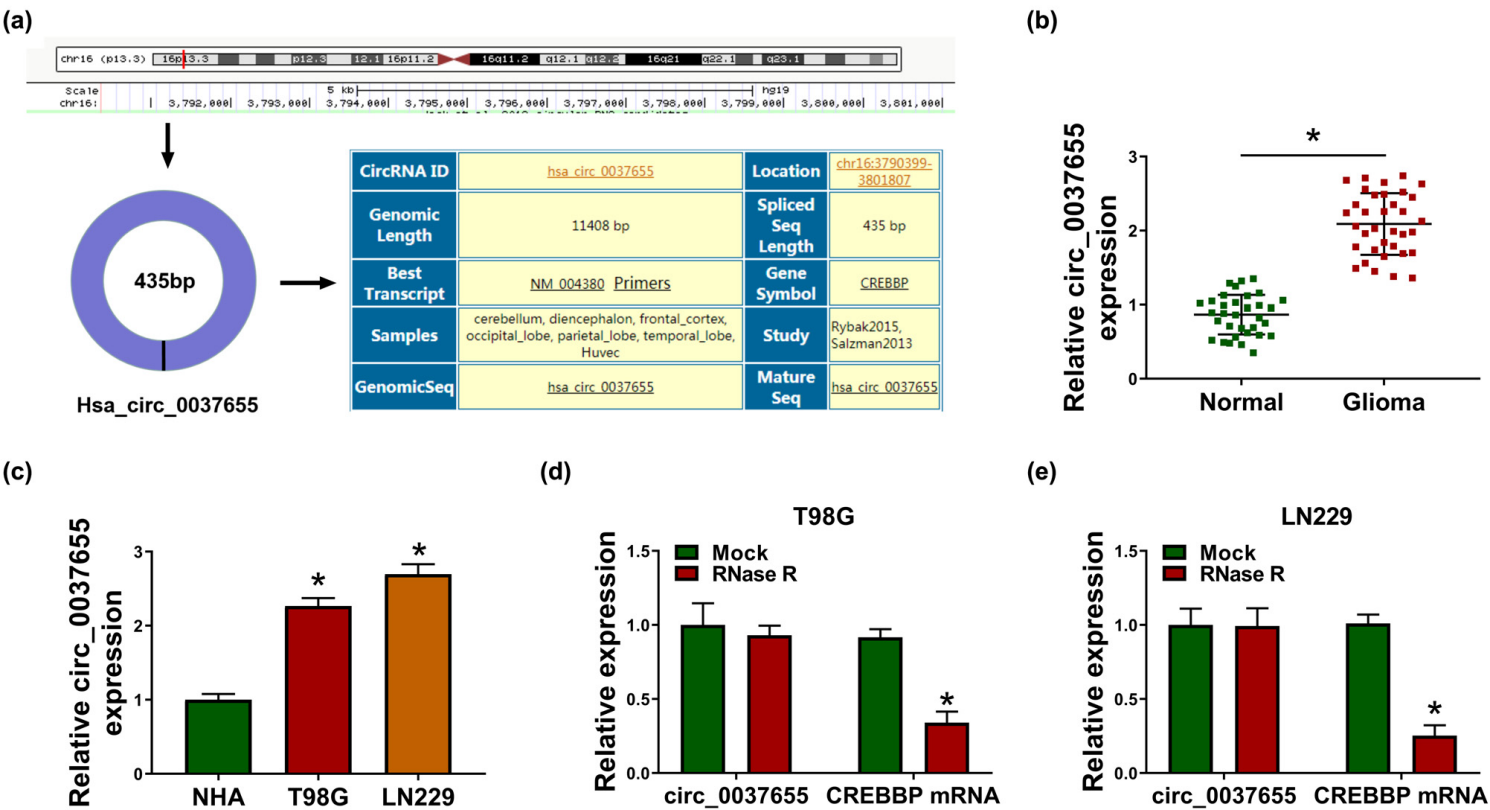
circ_0037655 was dysregulated with a high level in glioma samples and cells. (a) The genetic information of circ_0037655 in circinteractome. (b and c) circ_0037655 expression in tissue samples (normal and glioma tissues) and cell lines (NHA, T98G and LN229) was analyzed by qRT-PCR assay. (d and e) The qRT-PCR was used for the examination of circ_0037655 and CREBBP mRNA after treatment of RNase R in total RNA from T98G (d) and LN229 (e) cells. *P < 0.05.

circ_0037655 (hsa_circ_0037655) originates from CREBBP gene with the location at chr16:3790399-3801807 and the spliced sequence length of 435 bp, according to the online circinteractome (Figure 1a). After the qRT-PCR analysis for circ_0037655 expression, we found that its upregulation was conspicuous in glioma tissue specimens (Figure 1b) and cells (T98G and LN229) (Figure 1c) contrasted to these normal brain tissues and NHA cell line. In comparison with the obvious downregulation of CREBBP mRNA expression after RNase R treatment, circ_0037655 level was unchanged and highly stable (Figure 1d and e). It has been proved that circ_0037655 was highly expressed in glioma.

### 3.2 Downregulating circ_0037655 inhibited tumorigenesis and metastasis in glioma

**Figure 2:**
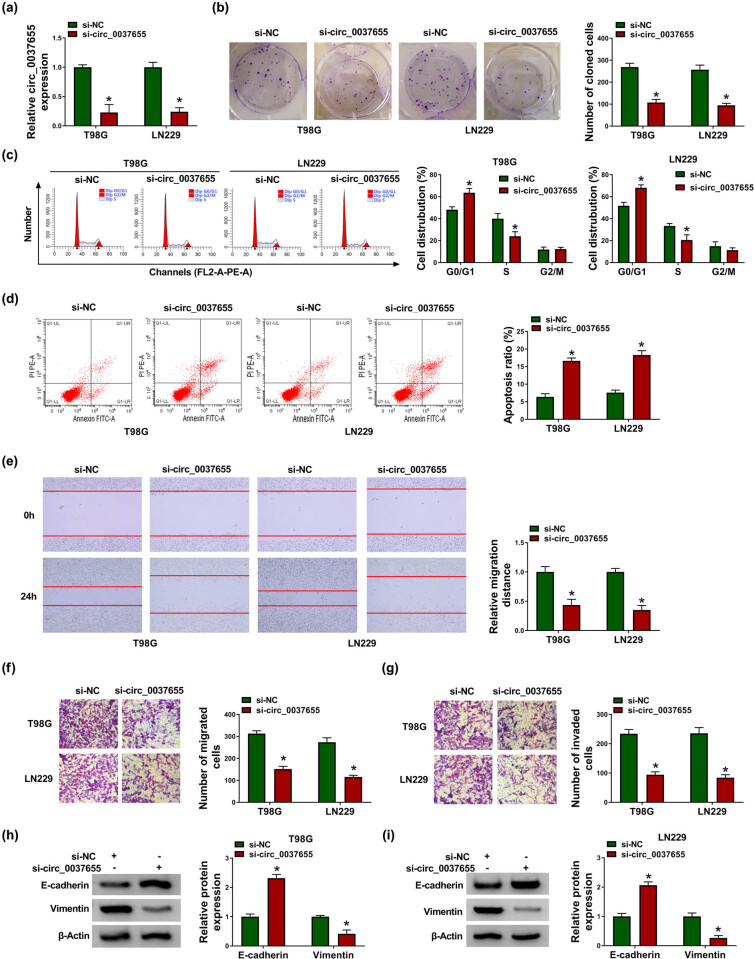
Downregulating circ_0037655 inhibited tumorigenesis and metastasis in glioma. Si-NC and si-circ_0037655 were, respectively, transfected into T98G and LN229 cells. (a) The detection of circ_0037655 was performed by qRT-PCR. (b) Colony formation assay was applied to determine cell proliferation. (c and d) Cell cycle (c) and apoptosis (d) were measured by flow cytometry. (e) Cell migration was assessed by scratch assay. (f and g) Transwell assay was conducted for analyzing cell migration (f) and invasion (g). (h and i) E-cadherin and Vimentin protein levels were examined via western blot. *P < 0.05.

In T98G and LN229 cells, transfection of si-circ_0037655 induced the 70% downregulation of circ_0037655 expression relative to the transfection of si-NC (Figure 2a). The transfection of si-circ_0037655 (compared to si-NC transfection) significantly decreased the number of cloned cells by performing colony formation assay (Figure 2b), showing that knockdown of circ_0037655 repressed glioma cell proliferation. Flow cytometry after the introduction of si-circ_0037655 manifested that cell transition from the G0/G1 phase to the S phase was blocked (Figure 2c) but cell apoptotic rate was increased by about 2.5-fold changes (Figure 2d), in comparison to the si-NC group. The migratory abilities of T98G and LN229 cells in the scratch assay were distinctly reduced with the downregulation of circ_0037655 (Figure 2e). The results of transwell assay suggested that the inhibitory influences on cell migration (Figure 2f) and invasion (Figure 2g) were caused by silencing the expression of circ_0037655. Epithelial–mesenchymal transition (EMT) is an important biological process driving cell metastasis [22]. E-cadherin (anti-EMT marker) protein expression was upregulated and Vimentin (EMT-promoting marker) was downregulated in the si-circ_0037655 group compared with the si-NC group (Figure 2h and i), implying that circ_0037655 inhibition inactivated the EMT process. All in all, glioma formation and metastasis *in vitro* were hampered by the downregulation of circ_0037655.

### 3.3 miR-1229-3p was an miRNA target of circ_0037655

**Figure 3:**
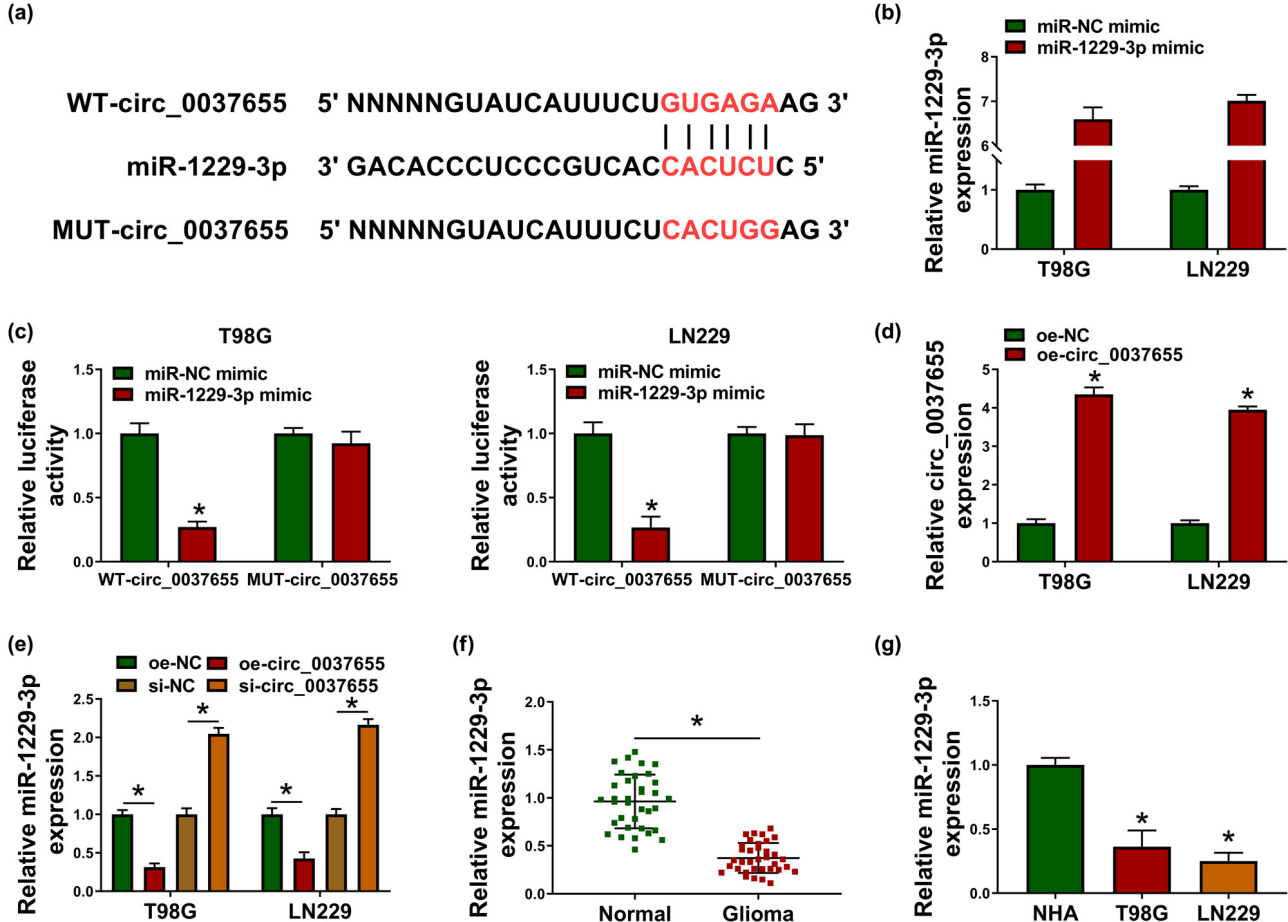
miR-1229-3p was an miRNA target of circ_0037655. (a) Circinteractome presented the site binding between circ_0037655 and miR-1229-3p. (b) The miR-1229-3p expression was assayed using qRT-PCR after transfection of miR-NC mimic or miR-1229-3p mimic. (c) The interaction between circ_0037655 and miR-1229-3p was analyzed by dual-luciferase reporter assay. (d) The overexpression efficiency of oe-circ_0037655 was evaluated by qRT-PCR. (e) After transfection of oe-NC, oe-circ_0037655, si-NC or si-circ_0037655, miR-1229-3p expression determination was carried out via qRT-PCR. (f and g) The expression analysis of miR-1229-3p in glioma tissues (f) and cells (g) was carried out by qRT-PCR. *P < 0.05.

Through the search and prediction by circinteractome (https://circinteractome.nia.nih.gov/), the binding sites were found between the sequences of circ_0037655 and miR-1229-3p (Figure 3a). The overexpressed effect of miR-1229-3p mimic on the expression of miR-1229-3p was affirmed by qRT-PCR (Figure 3b), then dual-luciferase reporter assay demonstrated that miR-1229-3p mimic interacted with WT-circ_0037655 to result in the inhibition of luciferase activity but it could not interact with MUT-circ_0037655 to affect the luciferase activity (Figure 3c). The qRT-PCR result exhibited the three-fold changes of circ_0037655 upregulation by transfection of oe-circ_0037655 contraposed to oe-NC transfection (Figure 3d). About the effect of circ_0037655 on miR-1229-3p level, circ_0037655 overexpression was found to downregulate the expression of miR-1229-3p while circ_0037655 knockdown induced the upregulation of miR-1229-3p (Figure 3e). In addition, there was a lower level of miR-1229-3p in glioma tissues (Figure 3f) and cells (Figure 3g) than that in normal tissues and cells. The target relationship of circ_0037655 to miR-1229-3p was confirmed.

### 3.4 miR-1229-3p inhibitor assuaged the si-circ_0037655-induced glioma progression inhibition

**Figure 4:**
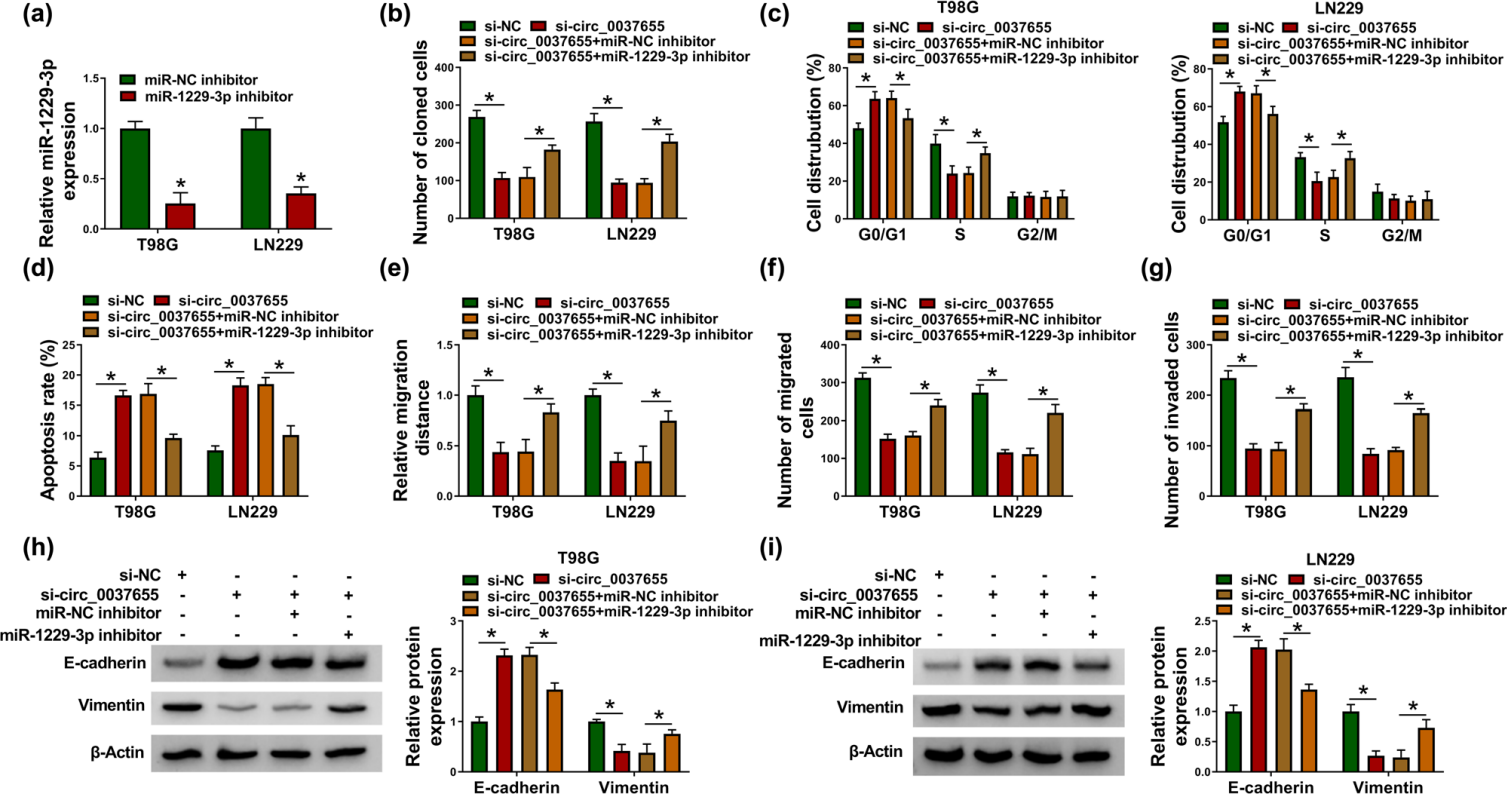
miR-1229-3p inhibitor mitigated the si-circ_0037655-induced glioma progression inhibition. (a) The qRT-PCR was implemented for analyzing the transfection efficiency of miR-1229-3p inhibitor. (b–i) In T98G and LN229 cells transfected with si-NC, si-circ_0037655, si-circ_0037655 + miR-NC inhibitor or si-circ_0037655 + miR-1229-3p inhibitor, cellular behaviors were performed by colony formation assay for cell proliferation (b), flow cytometry for cell cycle (c) and apoptosis (d), scratch assay for cell migration (e), transwell assay for migration and invasion (f and g), and western blot for EMT-associated protein detection (h and i). *P < 0.05.

Given the negative regulation of circ_0037655 on miR-1229-3p, T98G and LN229 cells were co-transfected with si-circ_0037655 and miR-1229-3p inhibitor to explore whether miR-1229-3p was associated with the function of si-circ_0037655. As shown in Figure 4a, miR-1229-3p expression inhibition was successfully evoked by miR-1229-3p inhibitor compared with the miR-NC inhibitor group. Cellular analysis for proliferation (Figure 4b), cell cycle (Figure 4c) and apoptosis (Figure 4d) displayed that the effects triggered by si-circ_0037655 were offset by miR-1229-3p inhibitor. The suppressive influences of si-circ_0037655 on cell migration (Figure 4e and f), invasion (Figure 4g) and EMT (Figure 4h and i) were also reversed after the transfection of miR-1229-3p inhibitor. These results suggested that glioma progression inhibition by knockdown of circ_0037655 was related to the upregulation of miR-1229-3p.

### 3.5 ITGB8 was a target gene in the downstream of miR-1229-3p

**Figure 5:**
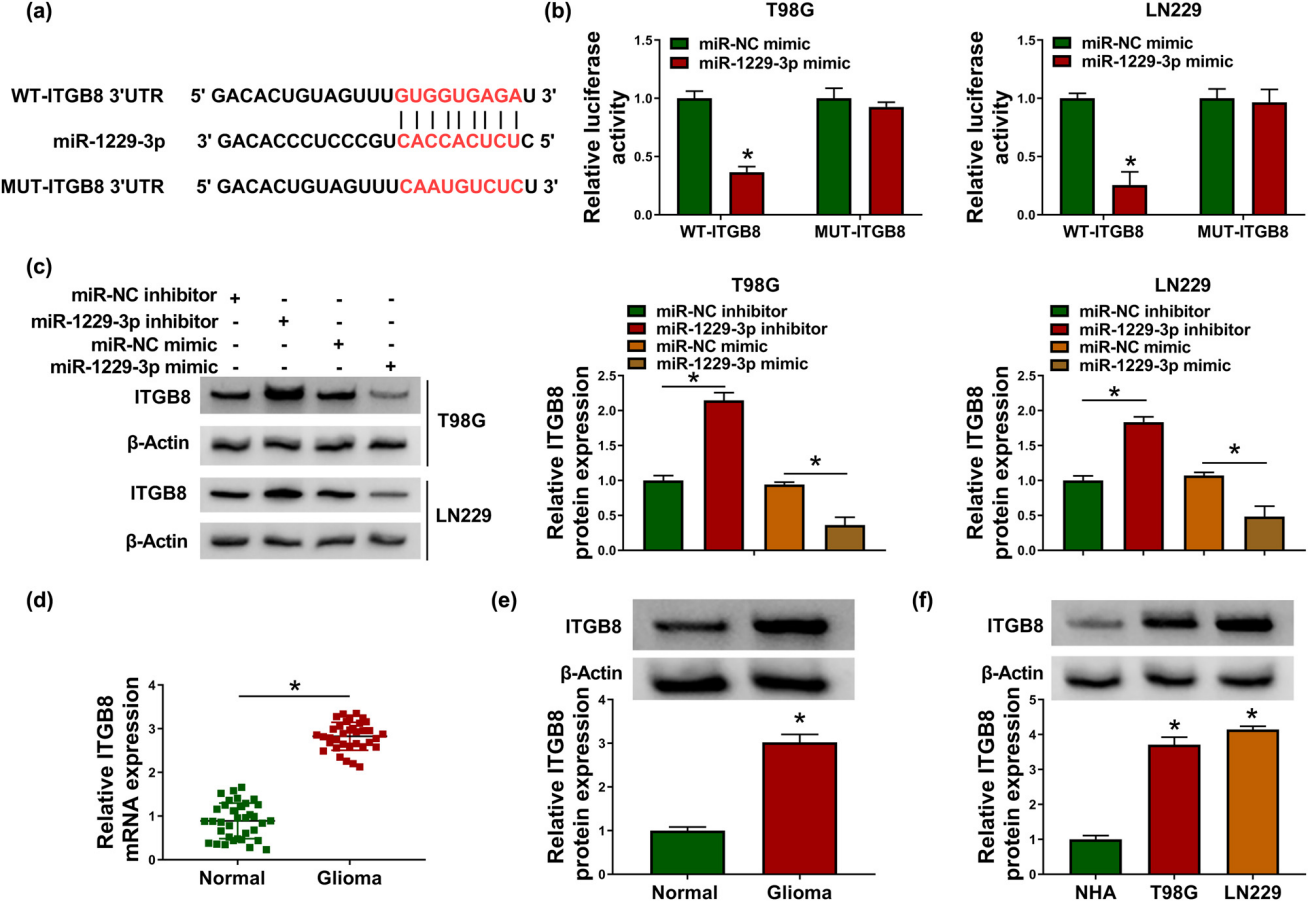
ITGB8 was a target gene in the downstream of miR-1229-3p. (a) The binding sites of miR-1229-3p in ITGB8 3′-UTR were analyzed by Targetscan. (b) Dual-luciferase reporter assay was conducted to verify that ITGB8 could combine with miR-1229-3p. (c) Western blot was exploited for protein level detection of ITGB8 in T98G and LN229 cells with transfection of miR-NC inhibitor, miR-1229-3p inhibitor, miR-NC mimic or miR-1229-3p mimic. (d and e) ITGB8 mRNA and protein levels in normal and glioma tissues were measured via qRT-PCR and western blot. (f) The analysis of ITGB8 protein expression in glioma cells was performed by western blot. *P < 0.05.

Targetscan (http://www.targetscan.org/) predicted that ITGB8 3′-UTR sequence contained the potential binding sites for miR-1229-3p (Figure 5a). The binding between miR-1229-3p and ITGB8 was validated by the miR-1229-3p mimic-induced luciferase signal inhibition of WT-ITGB8 plasmid in T98G and LN229 cells (Figure 5b). Western blot showed that the ITGB8 protein level of the miR-1229-3p inhibitor group was higher than that of the miR-NC inhibitor group and miR-1229-3p overexpression incurred the downregulation of ITGB8 (Figure 5c). The upregulation of ITGB8 mRNA and protein was found in glioma tissues (Figure 5d and e), as well as the same phenomenon of ITGB8 protein expression in T98G and LN229 cells in contrast with NHA cells (Figure 5f). ITGB8 was a veritable target of miR-1229-3p.

### 3.6 ITGB8 inhibition was accountable for the tumor-inhibitory role of miR-1229-3p in glioma cells

**Figure 6:**
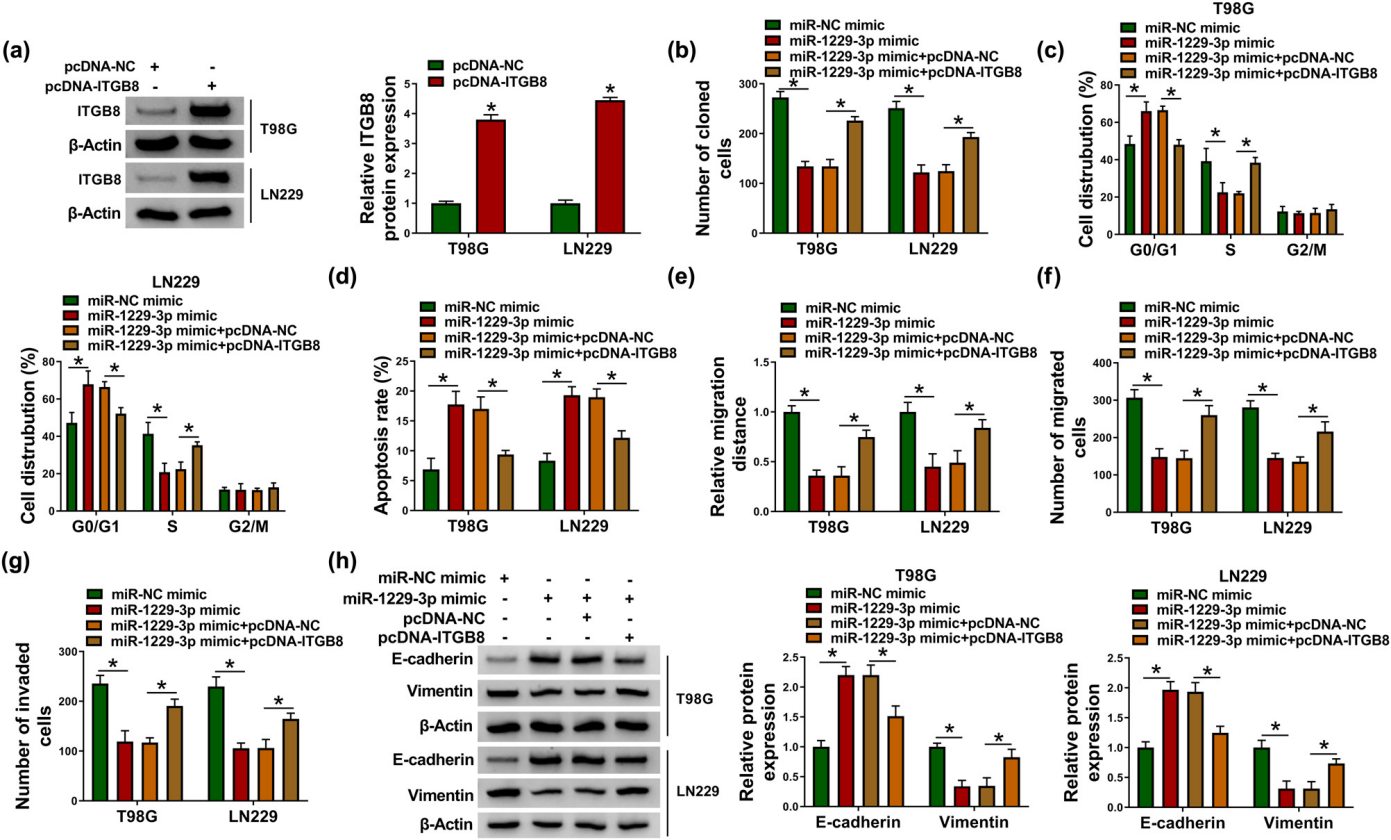
ITGB8 inhibition was accountable for the tumor-inhibitory role of miR-1229-3p in glioma cells. (a) ITGB8 protein detection was completed via western blot in pcDNA-NC and pcDNA-ITGB8 transfection groups. (b–h) Cell proliferation by colony formation assay (b), cell cycle (c) and apoptosis (d) by flow cytometry, cell migration by scratch assay (e), migration and invasion by transwell assay (f and g), and EMT analysis by western blot (h) were performed after transfection of miR-NC mimic, miR-1229-3p mimic, miR-1229-3p mimic + pcDNA-NC or miR-1229-3p mimic + pcDNA-ITGB8. *P < 0.05.

ITGB8 protein expression was greatly upregulated after transfection of pcDNA-ITGB8 in T98G and LN229 cells compared with pcDNA-NC transfection (Figure 6a). The subsequent assays revealed that miR-1229-3p overexpression led to cell proliferation repression (Figure 6b), cell cycle arrest (Figure 6c) and apoptosis acceleration (Figure 6d) in T98G and LN229 cells, whereas the upregulation of ITGB8 weakened these effects. Also, miR-1229-3p mimic was shown to suppress cell migration (Figure 6e and f), invasion (Figure 6g) and EMT process (Figure 6h) via reducing the expression of ITGB8. Thus, ITGB8 downregulation was accountable for the tumor-inhibitory role of miR-1229-3p in glioma.

### 3.7 ITGB8 was regulated by the circ_0037655/miR-1229-3p axis in glioma cells

**Figure 7:**
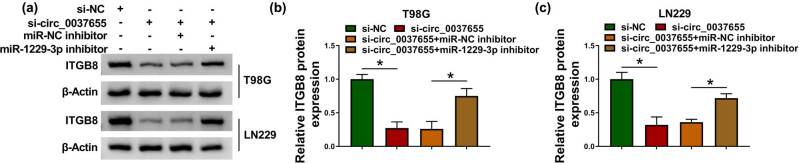
ITGB8 was regulated by the circ_0037655/miR-1229-3p axis in glioma cells. (a–c) ITGB8 protein expression examination was carried out via western blot in T98G and LN229 cells transfected with si-NC, si-circ_0037655, si-circ_0037655 + miR-NC inhibitor or si-circ_0037655 + miR-1229-3p inhibitor. *P < 0.05.

Western blot was used for the protein expression analysis of ITGB8 in T98G and LN229 cells transfected with si-NC, si-circ_0037655, si-circ_0037655 + miR-NC inhibitor or si-circ_0037655 + miR-1229-3p inhibitor (Figure 7a). As the data in Figure 7b and c, ITGB8 protein expression was declined in the si-circ_0037655 group relative to the si-NC group while this inhibition was partly abolished after the co-transfection of si-circ_0037655 and miR-1229-3p inhibitor. circ_0037655 could regulate the ITGB8 level via targeting miR-1229-3p.

### 3.8 Glioma tumorigenesis *in vivo* was retarded by the silence of circ_0037655 via the miR-1229-3p/ITGB8 axis

**Figure 8:**
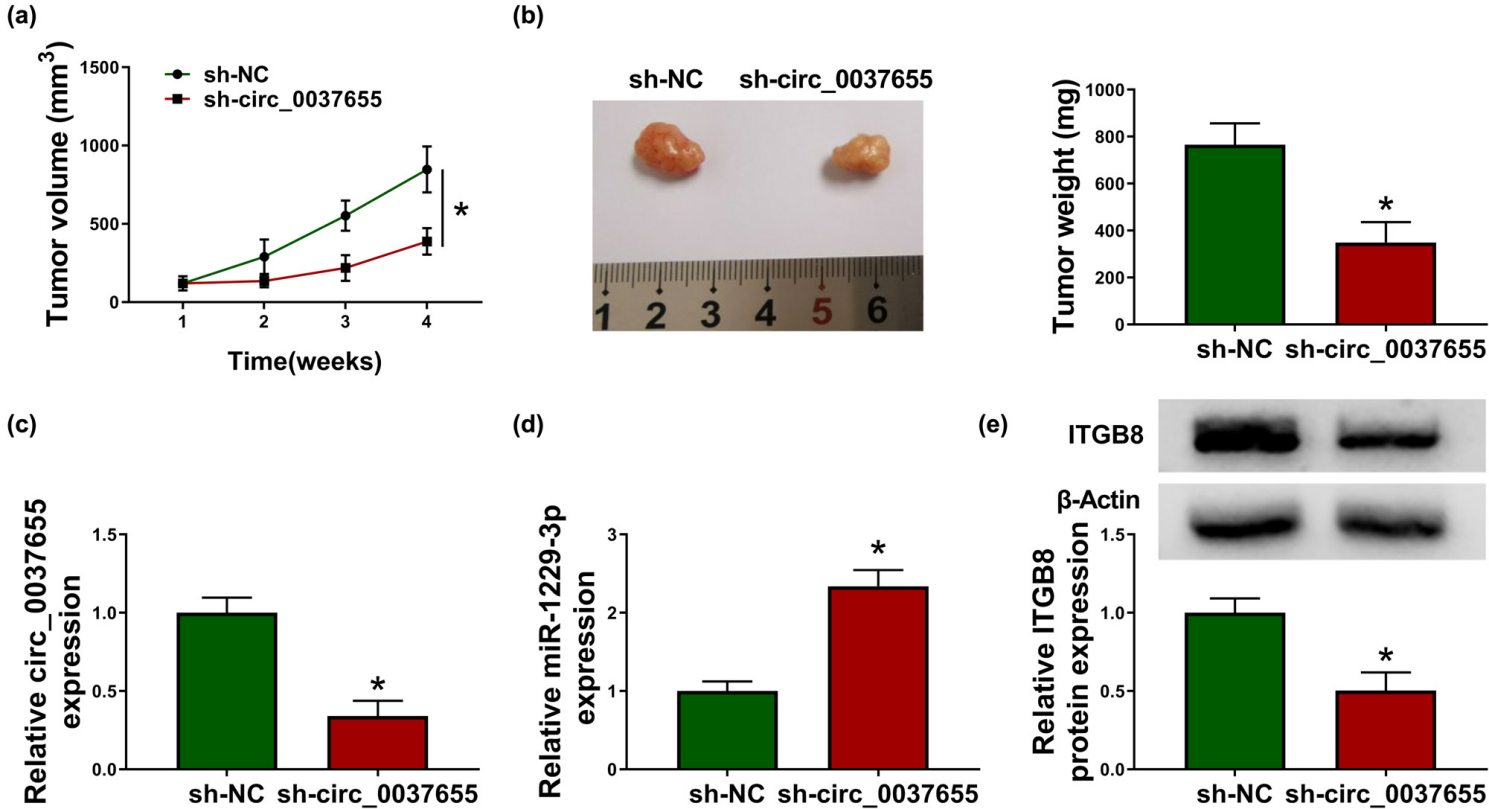
Glioma tumorigenesis *in vivo* was retarded by the silence of circ_0037655 via the miR-1229-3p/ITGB8 axis. (a and b) Tumor volumes in sh-NC and sh-circ_0037655 groups were determined weekly (a) and tumors were weighed after tumor excision (b). (c and d) The qRT-PCR was employed to assay the levels of circ_0037655 (c) and miR-1229-3p (d). (e) Protein expression of ITGB8 was detected by western blot. *P < 0.05.

Xenograft models were established in mice. Through the observation of 4 weeks, tumor volume (Figure 8a) and weight (Figure 8b) in the sh-circ_0037655 group were exhibited to be decreased in contrast with the sh-NC group. By detecting the expression of circ_0037655 in tumor tissues, we found that its expression inhibition was markedly caused by sh-circ_0037655 in mice (Figure 8c). Downregulation of circ_0037655 also promoted the miR-1229-3p level (Figure 8d) and restrained ITGB8 protein expression (Figure 8e) in tumor tissues. Collectively, circ_0037655 could regulate the glioma tumorigenesis *in vivo* by sponging miR-1229-3p and regulating the expression of ITGB8.

## 4 Discussion

Glioma is a refractory disease because of the local recurrence and distal metastasis [23]. Functional circRNAs have been considered as important regulators in glioma research [24]. Our investigation found that circ_0037655 acted as a tumor-promoting factor in the progression of glioma by regulating the levels of miR-1229-3p and ITGB8, which indicated a different molecular mechanism of glioma.

circRNAs have become the research hotspots of tumors in recent years. circRNAs have many biological properties, such as ubiquitous expression, tissue/cell specificity, high conservation and high stability [25]. Our qRT-PCR analysis revealed that circ_0037655 was differentially upregulated in glioma and it was resistant to the digestion of RNase R. Mounting tumor studies have clarified that circRNAs could be used as diagnostic and therapeutic targets for medical development due to their regulatory functions [26,27]. For instance, circHIPK3 regulated cellular processes in ovarian carcinoma and acted as a tumor repressor [28]; circ_0055538 retarded the malignant biological behavior in oral squamous cell carcinoma as a potential therapeutic biomarker [29]; circ_0102049 contributed to osteosarcoma cell migration and invasion as a metastatic indicator [30]; circPTPRM was reported to accelerate cell proliferation and migration of hepatocellular carcinoma [31]. In this study, the functional analysis of circ_0037655 suggested that its downregulation induced significant inhibition of glioma cell growth and cell cycle progression. Also, the same effects were observed on cell migration, invasion and EMT process. The repression of the downregulated circ_0037655 on glioma formation and metastasis demonstrated that circ_0037655 might be an available therapeutic marker in glioma treatment, as previously stated [14].

Subsequently, miR-1229-3p was found as an miRNA target of circ_0037655. circ_0037655/miR-1229-3p interaction was affirmed by dual-luciferase reporter assay and circ_0037655 could negatively regulate the expression of miR-1229-3p. Moreover, the reversal of miR-1229-3p inhibitor for si-circ_0037655-mediated progression suppression in glioma cells implied that the regulation of circ_0037655 was ascribed to the sponge effect on miR-1229-3p in glioma at least in part. Recent research studies have exhibited that glioma progression was modulated by circHIPK3 via interacting with miR-124-3p [32], circ_0079593 via sponging miR-182 and miR-433 [33], and circ-POSTN via targeting miR-1205 [34]. The sponge influences of circRNAs on miRNAs might be the crucial mechanisms for circRNA functions in glioma.

Meanwhile, we found that miR-1229-3p could bind to 3′-UTR of ITGB8 to downregulate the level of ITGB8 in glioma cells. miR-1229-3p acted as a tumor inhibitor in the development of glioma through the inhibition of ITGB8 expression. Furthermore, circ_0037655 was shown to have positive effect on the ITGB8 expression via the negative regulation of miR-1229-3p. This circ_0037655/miR-1229-3p/ITGB8 axis is a novel discovery in glioma. The circRNA–miRNA–gene regulatory networks have largely found in previous exploration of circRNAs in glioma, such as circ_0074362/miR-1236-3p/HOXB7 [35], circMAN2B2/miR-1205/SA00A8 [36] and circ_0088732/miR-661/RAB3D [37]. Our experiments *in vivo* also indicated that circ_0037655 expression inhibition suppressed glioma growth via regulating the levels of miR-1229-3p and ITGB8. Hence, miR-1229-3p/ITGB8 axis partly determined the specific function of circ_0037655 in glioma.

The current study has certain limitations. First, this study is limited by small sample size and a larger number of clinical samples are needed to provide the support for our conclusion. Second, the circ_0037655/miR-1229-3p/ITGB8 axis needs to be validated *in vivo* by the reverted assays. Third, it is interesting to investigate whether circ_0037655 is related to the overall survival and disease-free survival rates in glioma patients. Last but not the least, the clinical application of circ_0037655 as a therapeutic target requires further exploration. For example, combined with nanotechnology, circ_0037655 inhibition can be used as an effective strategy to inhibit the glioma progression.

## 5 Conclusion

**Figure 9:**
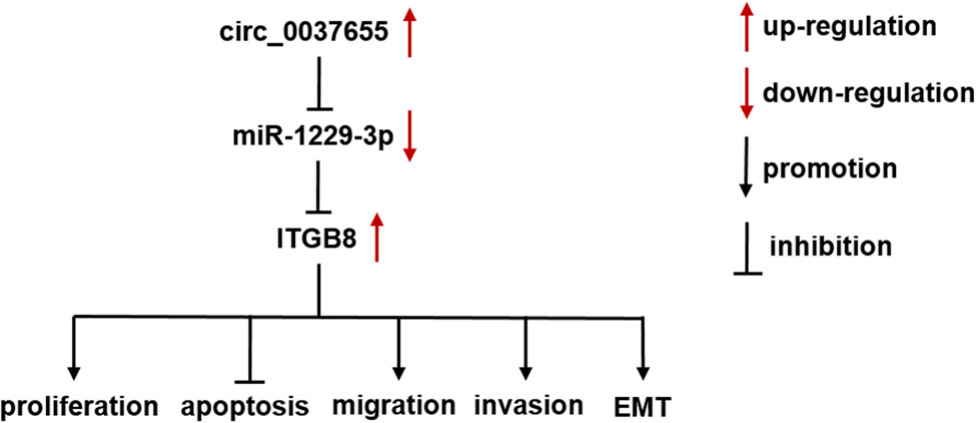
circ_0037655/miR-1229-3p/ITGB8 axis regulated the biological processes of glioma cells.

Taken together, the present study clarified that circ_0037655 sponged miR-1229-3p to promote the expression of ITGB8 to regulate the various cellular behaviors of glioma cells (Figure 9). It might lay the further theoretical foundation for circ_0037655 function in glioma, as well as the potential of circ_0037655 involving in the diagnosis and molecular therapy for glioma.
